# Niagara University Medical Department

**Published:** 1889-05

**Authors:** 


### *NIAGARA UNIVERSITY, MEDICAL DEPARTMENT.*

### Annual Convocation—Alumni Association.

The sixth annual session of lectures in this growing school of medicine closed in a blaze of success not before equaled in its history. The examinations of students for promotions from the lower to the higher grades, and also for recommendation by the faculty to the Board of Examiners for the degree of Doctor in Medicine, occupied two weeks. The result has demonstrated a most gratifying success for the improved system of graded instruction adopted by this school.

The Alumni Association met on Tuesday, April 9th, in the college building. The address of welcome, on behalf of the faculty, was delivered by Prof. Albert E. Persons, who tendered to the Alumni a cordial greeting, and in earnest words commended the labors of his colleagues in the cause of higher medical education. The President, Dr. C. C. Frederick, gave the annual address, in which he reviewed the work of the year, and implored the coöperation of the Alumni and friends of the college in its efforts to establish a much-needed reform in the system of medical instruction. The business meeting which followed resulted in the election of several new members. Drs. J. D. Flagg, John A. Miller, Eugene A. Smith, A. M. Ewing, and T. Haven Ross, of the medical faculty, were elected honorary members.

The following were elected officers for the ensuing year:

*President,* Geo. W. T. Lewis, M. D.; *First Vice-President,* Lawrence G. Hanley, M. D.; *Second Vice-President,* C. B. LeVan, M. D.; *Permanent Secretary,* Frank H. Potter, M. D.; *Secretary,* E. N. Pfohl, M. D.; *Treasurer,* H. D. Ingraham, M. D.; *Execu-*

*tive Board*, Drs. S. A. Dunham, A. E. Persons, and D. L. Redmond.

The meeting for the reading and discussion of papers was held in the lecture-room of the Y. M. C. A., and was attended by a large number of the medical men of the city and vicinity. The President, Dr. Frederick, presided, and introduced Prof. Herman Mynter, who read the first paper, on "Acute Infectious Osteo-Myelitis." The second paper was read by Dr. Hermann M. Biggs, Pathologist to the Carnegie Laboratory, New York, on "The Prevention and Principles of Treatment of Pulmonary Tuberculosis as based on its Etiology and Pathology." The third paper was read by Dr. Chas. A. L. Reed, of the Cincinnati College of Medicine and Surgery, and Cincinnati Polyclinic, on "The Gynecic Element in Psychiatry." The papers were able and the discussions which followed were pointed and interesting. Dr. Reed's paper appears in this issue of the Journal, and Dr. Biggs's will be published in the June number.

The commencement exercises were held in Association Hall in the evening, and were attended by an audience that filled it to its utmost capacity. Prof. John Cronyn, President of the medical faculty, presided and acted as master of ceremonies. The Chancellor of the University, the Rt. Rev. Stephen Vincent Ryan, D. D., conferred the degree of Doctor in Medicine on the following gentlemen, who had been recommended to the Trustees by the Board of Medical Examiners:

John M. Hewitt, Allegany, N. Y.; John T. Culhane, Victor, N. Y.; Frank B. Voght, Lockport, N. Y.; Alfred W. Bayliss, South Wales, N. Y.; Chas. A. Schladermundt, Buffalo, N. Y.; Edgar A. Forsyth, Buffalo, N. Y.; Joe. E. Widner, Buffalo, N. Y.; Walter John Riehl, Buffalo, N. Y.; Washington I. Hewitt, Leola, South Dakota.

The address to the graduates was delivered by Dr. R. Stansbury Sutton, of Pittsburgh, Pa., and was full of rich thought beautifully expressed, on the claims of legitimate and scientific medicine on the novitiate and from the community. Rarely have we listened to an address more eloquent and practical.

The banquet followed at the Genesee. Dr. Frank H. Potter acted as toastmaster, and called on the Rev. Father Grace, who responded to the toast, "The Niagara University," Dr. C. C. Frederick on the "Alumni Association," Dr. C. A. L. Reed replied to the "Wild and Wooly West," "The Clergy," by Rev. E. E. Chivers, "The Law Department," by Chas. P. Norton, "Parasitic Diseases of the Microbe," by Dr. H. M. Biggs, "The Press," by F. H. Severance, "Women," by Dr. R. S. Sutton, "The Class of 1889," by Dr. J. M. Hewitt.

"The Lamented and Honored Dead" was responded to by Prof. Simeon Tucker Clark. This sentiment, consisting of an original poem, was offered in memory of the late Profs. Davidson and Campbell, members of the faculty, and also collaborators in the work of the JOURNAL.

The entire exercises, from the commencement of the examinations of the students until their conclusion, were of a high character, reflecting great credit on the college, and giving encouragement to its faculty in their work. This city has seldom witnessed a meeting conducted with such rare ability. The faculty and alumni may well be congratulated on its success.

We trust that the next meeting may be of like professional and social interest and value.

